# Learning of anticipatory responses in single neurons of the human medial temporal lobe

**DOI:** 10.1038/ncomms9556

**Published:** 2015-10-09

**Authors:** Leila Reddy, Marlene Poncet, Matthew W. Self, Judith C. Peters, Linda Douw, Edwin van Dellen, Steven Claus, Jaap C. Reijneveld, Johannes C. Baayen, Pieter R. Roelfsema

**Affiliations:** 1 Université de Toulouse, Centre de Recherche Cerveau et Cognition, Université Paul Sabatier, 31052 Toulouse, France; 2 CNRS, UMR 5549, Faculté de Médecine de Purpan, 31052 Toulouse, France; 3 Department of Vision and Cognition, Netherlands Institute for Neuroscience (KNAW), 1105 BA Amsterdam, The Netherlands; 4 Neuroimaging & Neuromodeling Group, Netherlands Institute for Neuroscience, Royal Netherlands Academy of Arts and Sciences (KNAW), Meibergdreef 47, 1105 BA Amsterdam, The Netherlands; 5 Department of Cognitive Neuroscience, Faculty of Psychology and Neuroscience, Maastricht University, 6200 MD Maastricht, The Netherlands; 6 Department of Anatomy and Neurosciences, VU University Medical Center, Van der Boechorststraat 7, 1081 BT Amsterdam, The Netherlands; 7 Department of Radiology, Athinoula A. Martinos Center for Biomedical Imaging/Massachusetts General Hospital, 149 13th Street, Charlestown, Massachusetts 02129, USA; 8 Department of Psychiatry, Brain Center Rudolf Magnus, University Medical Center Utrecht, PO Box 85500, 3508 GA Utrecht, The Netherlands; 9 Department of Neurophysiology, VU University Medical Center, PO Box 7057, 1007 MB Amsterdam, The Netherlands; 10 Stichting Epilepsie Instellingen Nederland, Achterweg 5, 2103 SW Heemstede, The Netherlands; 11 Department of Neurology, VU University Medical Center, PO Box 7057, 1007 MB Amsterdam, The Netherlands; 12 Department of Neurosurgery, VU University Medical Center, PO Box 7057, 1007 MB Amsterdam, The Netherlands; 13 Department of Integrative Neurophysiology, Centre for Neurogenomics and Cognitive Research, Vrije Universiteit, 1081 HV Amsterdam, The Netherlands; 14 Psychiatry Department, Academic Medical Center, 1105 AZ Amsterdam, The Netherlands

## Abstract

Neuronal processes underlying the formation of new associations in the human brain are not yet well understood. Here human participants, implanted with depth electrodes in the brain, learned arbitrary associations between images presented in an ordered, predictable sequence. During learning we recorded from medial temporal lobe (MTL) neurons that responded to at least one of the pictures in the sequence (the preferred stimulus). We report that as a result of learning, single MTL neurons show asymmetric shifts in activity and start firing earlier in the sequence in anticipation of their preferred stimulus. These effects appear relatively early in learning, after only 11 exposures to the stimulus sequence. The anticipatory neuronal responses emerge while the subjects became faster in reporting the next item in the sequence. These results demonstrate flexible representations that could support learning of new associations between stimuli in a sequence, in single neurons in the human MTL.

## Abstract (web summary)

The medial temporal lobe (MTL) in the primate brain is known to alter its response when learning new information. Here, Reddy *et al.* record the activity of single neurons from human subjects, and show that learning a sequence of events can increase MTL neuronal firing rates in anticipation of the upcoming events.

## Graphical abstract

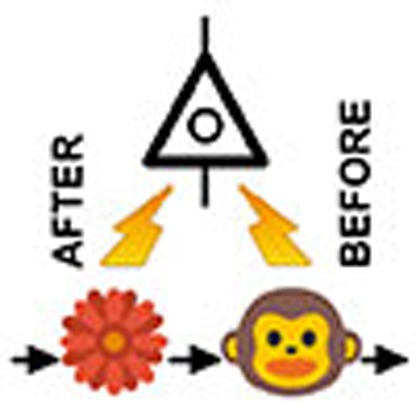

Adapting to our environment involves learning associations between stimuli that frequently occur together. These learned associations may become so firmly established that experiencing one stimulus can vividly evoke the other. Creating associations between different, initially unrelated stimuli relies on the medial temporal lobe (MTL)^1^. Previous studies have shown that single neurons in the monkey MTL signal learned associations by changing their response properties^2^,^3^,^4^,^5^,^6^,^7^. For example, neuronal responses to pairs of associated pictures become more similar to each other as a result of learning^2^, and significant neuronal activity is also observed during the delay period between presentations of two associated stimuli^5^. These learned responses are observed both when the animal has to learn a new rule, as well as when learning is incidental.

In humans as observed with functional magnetic resonance imaging (fMRI), rapid sequential associative learning has also been observed in the MTL. For example, MTL structures respond more strongly to structured versus unstructured sequences of visual stimuli^8^, and their activity depends on the statistical regularities of the sequence^9^,^10^,^11^. Fine-grained multi-voxel pattern analysis revealed that hippocampal and parahippocampal representations for associated objects become correlated to each other as a result of learning. Furthermore, while in most MTL regions the representations of associated objects become more similar during learning, in the CA3 region of the hippocampus sequence learning causes anticipatory shifts in activity^12^.

In this study we take advantage of the unique opportunity of recording from single neurons in the human MTL to study sequence learning at the single-neuron level. Subjects learned associations between visual stimuli presented in an ordered, predictable sequence. We report that sequence learning causes an increase in neuronal firing rates in anticipation of the neuron’s preferred stimulus (that is, a stimulus that would elicit a selective neuronal response when presented outside the sequence), within 11 trials of learning. These anticipatory responses could play an important role in predicting future events based on what has been recently learned.

## Results

### Learning procedure and behaviour

**Figure 1:**
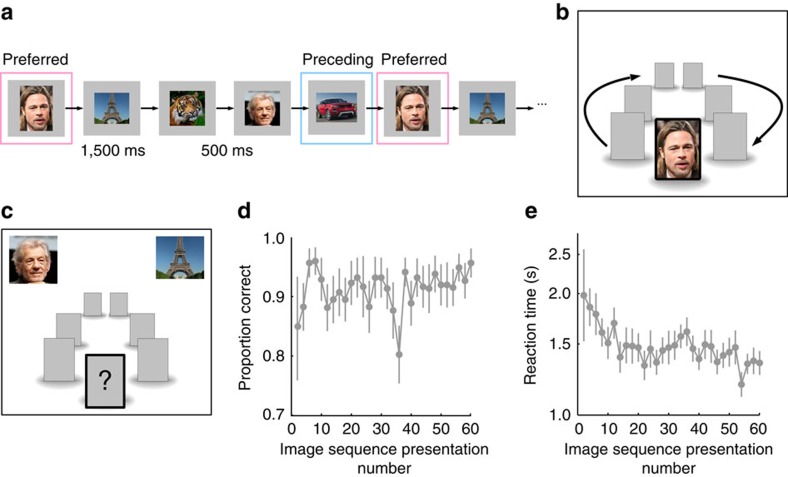
Learning task and behaviour. (**a**) During the AL sessions we presented a sequence of 5–7 images in a fixed order. The sequence contained one preferred image for a selective neuron identified in prior screening sessions. The image preceding the preferred image was always non-preferred for the recorded neuron. Each image was presented for 1.5 s followed by an ISI of 500 ms. The sequence was repeated 60 times. (**b**) Each image was presented at the centre of the screen while three placeholders (empty grey squares) were presented on either side. At the end of the image presentation period a placeholder replaced the central image and the placeholders moved in the clockwise direction for the duration of the ISI. At the end of the ISI the central placeholder was replaced by the next image in the sequence. (**c**) Twenty per cent of trials were test trials on which patients (*N*=8) saw two choice images and had to report which of the two would be the next image in the sequence. (**d**) Average accuracy and (**e**) average reaction times, shown on a log scale (to increase visibility), on test trials averaged across all subjects and sessions, as a function of image sequence presentation number (binned by groups of two sequence presentations). Error bars represent s.e.m. Accuracies reached their maximum by ∼8 sequence presentations. Reaction times decreased more progressively with sequence presentation number. Eiffel Tower, Benh Lieu Song, CC BY-SA 2.0. Ian McKellen, Gage Skidmore, CC BY-SA 3.0.

Eight subjects learned arbitrary associations between visual stimuli in 27 short associative learning (AL) sessions that lasted 10–14 min each. Each AL session consisted of 60 repetitions of a sequence of 5–7 images presented in a predetermined, predictable order, and subjects had to learn the order of the sequence. The images were displayed for 1,500 ms each, with an inter-stimulus interval (ISI) of 500 ms (Fig. 1a,b). We probed the learning process by including 20% of test trials where subjects had to predict the next image (Fig. 1c). Subjects learned the sequence rapidly, achieving >90% performance on test trials within six sequence presentations, while reaction times continued to decrease over a longer period (Fig. 1d,e). Each image sequence contained at least one ‘preferred’ image that drove one of our selective neurons (determined in independent screening sessions; see Methods). We ensured that the image immediately preceding the preferred image was always non-preferred for the recorded neuron.

### Electrophysiology

We recorded from 635 neurons in eight patients implanted with depth electrodes^13^. A total of 56 of these neurons were selective to one of the images presented in the screening sessions. In all, 42 of the selective neurons were in the hippocampus and parahippocampal cortex. These neurons are hereafter referred to as MTL neurons. In all, 14 of the selective neurons were located in the posterior temporal lobe. The quality of the recordings and spike sorting is shown in Supplementary Figs 1 and 2.

### Anticipatory responses in human MTL neurons

**Figure 2:**
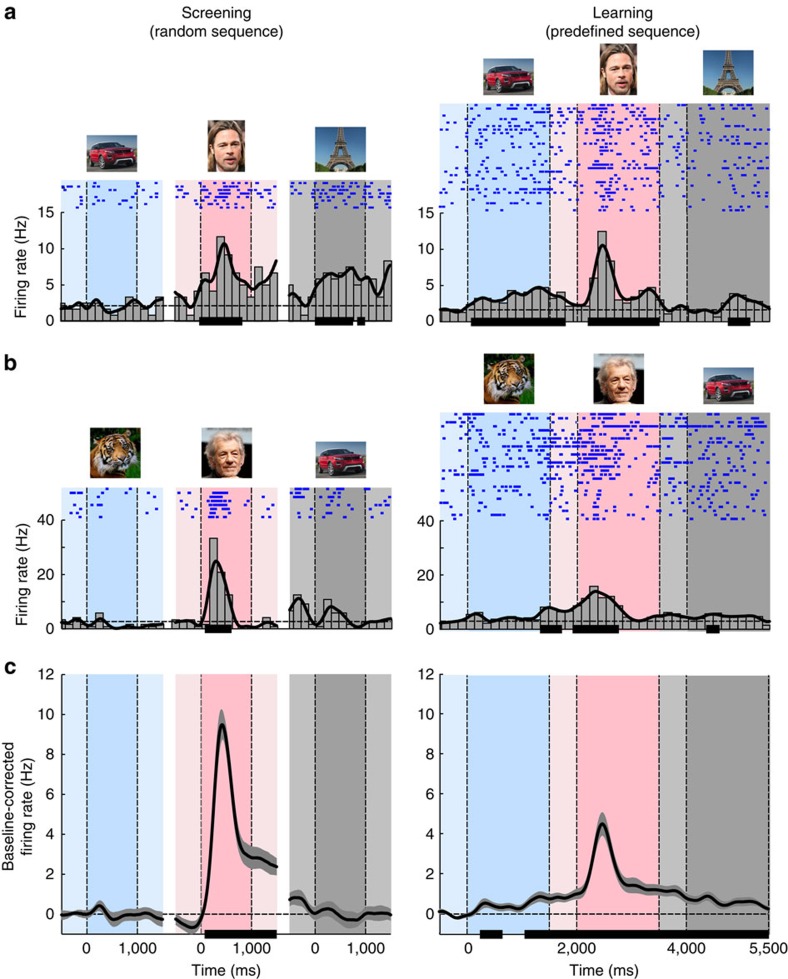
Anticipatory shifts in spiking activity during AL. (**a**) The left panel illustrates a screening session when the stimuli were presented in a randomized order. The right panel illustrates an AL session when the subject learned the order of the stimuli. The darker blue, pink and grey areas correspond to the presentations of the preceding, preferred and following stimuli. The lighter areas correspond to the ISIs. The dashed horizontal line is the average firing activity in the ISI window before the preceding stimulus. The solid black horizontal lines on the *x* axes represent time periods during which activity was significantly greater than the mean ISI response (that is, in the −500 to 0 ms window) before the preceding stimulus, continuously for at least 100 ms (paired *t*-test, *P*<0.05; see Methods). The spikes fired on individual trials are shown in the blue raster plots. This unit increased activity during the presentation of the preceding stimulus during AL, although this stimulus did not elicit a significant response during the screening session (left panel). (**b**) Another cell with increased activity during the ISI window between the preceding and preferred stimuli after learning. (**c**) Average activity of MTL neurons (*N*=42). Anticipatory activity before the preferred stimulus occurred during AL, but not during the screening sessions. Shaded area is the s.e.m. The dashed horizontal line at 0 is the baseline-corrected average ISI activity before the preceding stimulus. The solid black horizontal lines on the *x* axes represent time periods with activity significantly greater than in the ISI (−500 to 0 ms) before the preceding stimulus, for at least 300 ms. Note that the actual pictures used in the experiments are not shown here, as they were personal photographs from the patients. Eiffel Tower, Benh Lieu Song, CC BY-SA 2.0. Ian McKellen, Gage Skidmore, CC BY-SA 3.0.

In our first analysis we examined whether learning causes MTL neurons to start responding earlier in the sequence in anticipation of their preferred stimuli. Such an anticipatory response might occur during the ISI preceding the preferred stimulus or even during the presentation of the preceding stimulus itself. Figure 2a,b illustrates two example MTL neurons that increased their firing rates in anticipation of the preferred stimulus during AL. The cell shown in Fig. 2a was located in the hippocampus and showed significant activity for the preceding stimulus as a result of learning (right panel; paired *t*-test, *t*(30)=5.71; *P*<0.00001), whereas selectivity for this stimulus was absent during the screening session when the images were presented in a random order (left panel). The neuron in Fig. 2b was recorded in the parahippocampal cortex and showed significant anticipatory activity during the ISI (that is, the −500 to 0 ms window) just before the presentation of the preferred stimulus (paired *t*-test, *t*(26)=5.19; *P*<0.0001).

When considering the latency of the response, the activity of individual MTL neurons during screening sessions increased significantly above baseline 258±157 ms (mean±s.d. across individual cells) after the onset of the preferred stimulus. During AL, however, individual MTL neurons showed anticipatory activity that started 297±740 ms before the onset of the preferred stimulus (*t*(32)=−12.2; *P*<0.00001), or, equivalently, 1,703±740 ms after the onset of the preceding stimulus. The anticipatory activity was particularly evident when we averaged responses across all MTL cells (Fig. 2c; see Supplementary Fig. 3 for normalized responses). During AL, the average firing rate increased significantly above baseline 1,738 ms before the onset of the preferred stimulus, that is, even during the presentation of the preceding stimulus. These anticipatory effects specifically occurred during the presentation of the stimulus that preceded the neurons’ preferred stimulus and the subsequent ISI, but not for the non-preferred stimuli in the sequence (Supplementary Fig. 4). Similar effects were not observed for neurons recorded in the posterior temporal cortex (Supplementary Fig. 5). It can also be seen that the peak responses elicited by the preferred stimulus were weaker in the AL sessions than in the screening sessions. This decrease in visually driven activity is presumably caused by an adaptation effect caused by the frequent presentation of a few images during AL, as has also been observed in previous studies^14^,^15^ (Supplementary Fig. 6). Thus, the increase of activity during the preceding stimulus and ISI was accompanied by a decrease in the visual response, as if a fraction of the omitted visually driven spikes now occurred at an earlier point in time.

### Time course of anticipatory responses

**Figure 3:**
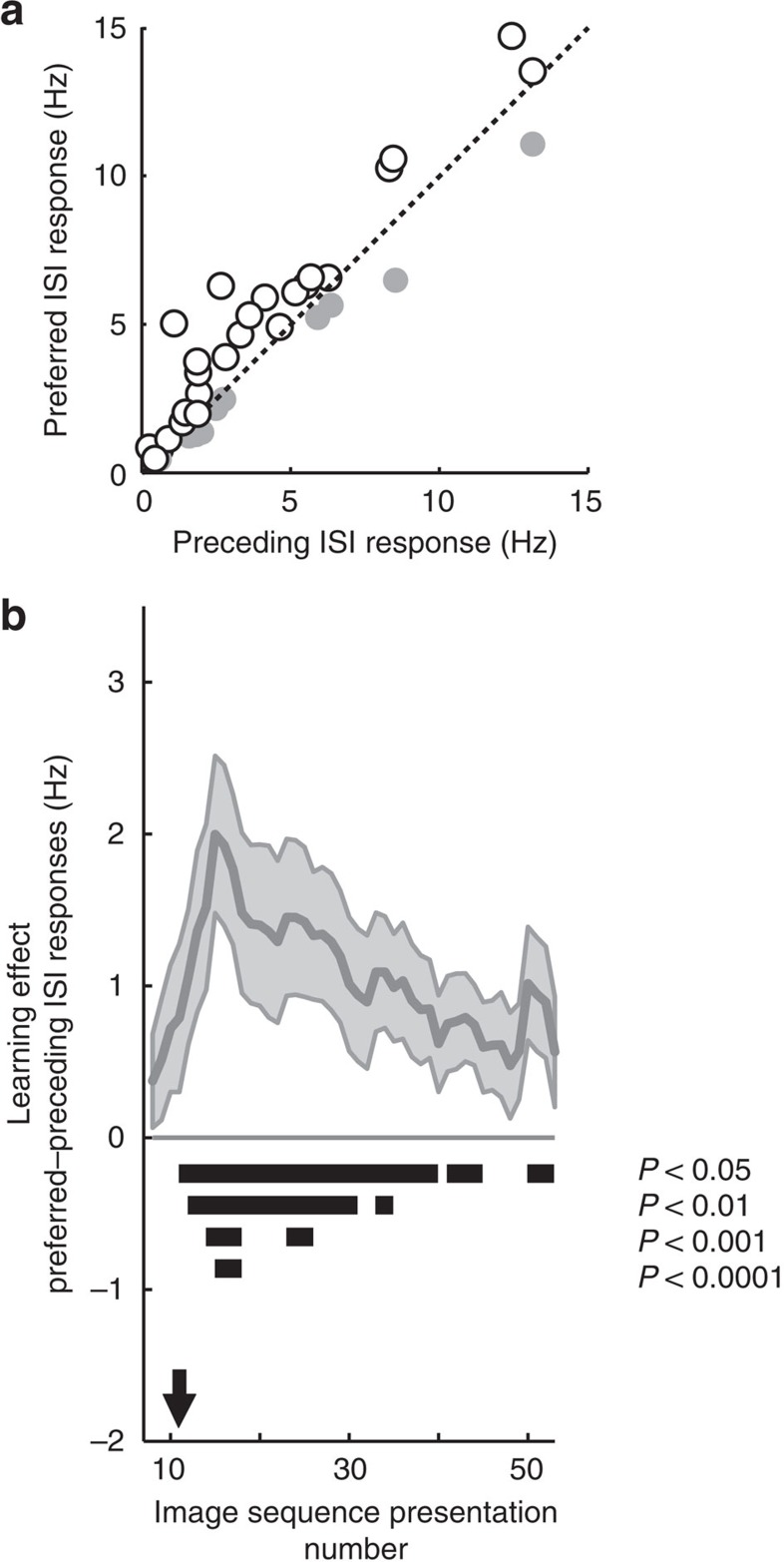
Time course of anticipatory responses in MTL cells (*N*=42). (**a**) Comparison of firing rate during the ISI periods (that is, −500 to 0 ms before stimulus onset) before the preferred and preceding stimuli during AL. Sixty-nine per cent of MTL cells (white circles) showed higher firing rates in the ISI before preferred stimuli (paired *t*-test, *P*<0.05). The grey circles correspond to the remaining cells. (**b**) The strength of the predictive activity calculated with a running average of 15 trials, where on each trial *n* we considered the difference between the preferred and preceding ISIs based on a moving average of trials *n*−7 to *n*+7. The solid black horizontal lines indicate which trials showed a significant learning effect, as computed by a non-parametric bootstrap procedure over 100,000 iterations. The black arrow indicates the eleventh trial, on which the difference between the preferred and preceding ISIs was first significant (*P*<0.05, non-parametric bootstrap procedure). See also Supplementary Fig. 8.

To assess the statistical significance of the learning-related increased firing rates during the ISI (500-ms window preceding stimulus onset) before the preferred stimulus, we compared it with the ISI activity before the preceding stimulus for the MTL cells (Fig. 3a and Supplementary Fig. 7). A two-way random effect analysis of variance (ANOVA) of ISI type (preferred versus preceding) × session (AL versus screening) revealed a significant interaction (*F*(1,123)=15.6, *P*<0.0005) because the ISI firing rate before the preferred stimulus was only higher in the AL sessions (*post hoc* paired test, *t*(41)=3.3; *P*<0.005). Sixty-nine per cent of neurons (*χ*^2^-test against 50%, *P*<0.05) showed an effect in this direction, and over the group of all neurons the average increase in firing was 37±13%.

MTL neurons thus signal new associations by increasing activity to previously non-preferred stimuli and during the ISI, in anticipation of their preferred stimuli. We next asked how these anticipatory changes evolve during learning. We defined the anticipatory learning effect as the difference between the mean activity during the ISI period (500-ms window before stimulus onset) before the preferred stimulus and the mean activity during the ISI period before the preceding stimulus. This anticipatory activity of single neurons on individual trials was variable (since it corresponds to an increase in baseline firing rates, in the absence of any stimulus presentation). Therefore, we used a 15-trial sliding window average in which, for each stimulus presentation *n*, we considered the difference between the preferred and preceding ISIs, based on a moving average of the *n−*7th to the *n*+7th stimulus presentations (Fig. 3b). The anticipatory learning effect became significant starting at the eleventh stimulus presentation (that is, averaging across presentations 4–18; *P*<0.05, non-parametric bootstrap procedure). The effect of performing the sliding window average for various numbers of trials is shown in Supplementary Fig. 8.

Subjects’ ability to learn the image sequence was evaluated on test trials that were interspersed throughout the AL sessions. On test trials, subjects saw two pictures and had to choose the one that matched the next one of the sequence (Fig. 1c). We chose this two-alternative force choice procedure as a sensitive measure of early learning, which might be expected to precede the ability of the subject to actively predict the next item of the sequence (for example, ref. 16). The accuracy on these test trials increased quickly and reached its maximum after ∼8 sequence presentations (Fig. 1d), confirming the sensitivity of the two-alternative forced choice test to early learning. Interestingly, the increase in accuracy on test trials preceded the predictive neuronal activity in the MTL, which occurred after ∼11 sequence presentations. It seems likely that this lag in predictive activity in the MTL is related to a delay in the subjects’ ability to actively predict the next item of the sequence. Although we did not require subjects to freely recall the next item, we considered the possibility that recall might also improve performance in a forced choice test by decreasing reaction time. Indeed, the subjects’ reaction times in the test trials continued to decrease for several trials after the appearance of predictive activity in the MTL (Fig. 1e). Furthermore, the change in behavioural reaction times on the test trials was significantly correlated with the time course of the learning effect at the neuronal level, as determined by a linear regression analysis of the neuronal learning effect (Fig. 3b) versus the reaction times (*r*^2^=0.18, *P*<0.0009).

Even though the correlation analysis above suggests that the behavioural and neuronal learning time courses behave similarly, the anticipatory learning effect also appears to peak relatively early, around the fifteenth trial, before slowly decaying. This might reflect the transient involvement of MTL structures during the early stages of sequence learning, after which learning is consolidated in other brain areas^1^.

To investigate whether the short episode of learning caused longer-lasting changes in neuronal tuning, we investigated whether the enhanced response elicited by the preceding stimulus persisted when the subjects passively viewed the same stimuli presented in random order (Supplementary Fig. 9). Passive viewing abolished the enhanced response to the stimulus that had preceded the preferred stimuli during sequence learning, implying that the short training epoch did not have an enduring influence on neuronal tuning outside the context of the sequence-learning task.

## Discussion

We here found that when subjects learn an ordered sequence, neurons in their MTL change their activity. The neurons start showing anticipatory changes in firing activity after 11 exposures to the image sequence. This predictive activity may be related to the recall of the next stimulus in the sequence and it implies the formation of a new association between the preferred stimulus and the one preceding it. Interestingly, these predictive responses only occurred within the context of the sequence task but not when subjects saw the same images in a random sequence outside the sequence-learning task.

The predictive responses in the human MTL are reminiscent of anticipatory activity of place cells^17^ in the rodent hippocampus. In a sequentially ordered spatial environment, place cells also start to fire progressively earlier within a few crossings of the track, thereby encoding the future position of the animal^18^,^19^,^20^. These place cell responses have been proposed to play a general role in encoding spatial and non-spatial events in temporally organized sequences^21^,^22^,^23^, thereby endowing the MTL with the ability to code for the temporal structure of longer sequences (for example, the visual stimuli in our learning task or spatial locations in a maze). According to this view, the anticipatory responses in the MTL could serve as the ‘glue’ that links successive events together into longer episodes.

The anticipatory responses could arise from at least two mechanisms. First, the new association could cause activity to spread between the representations of the preceding and preferred stimuli (for example, via pattern completion). Second, as a result of learning the now associated stimuli could be encoded as a single chunk^24^, and learning could cause neurons to acquire a new selectivity for this combination of the preceding and preferred stimuli. The high temporal resolution of single-cell recordings provides a unique opportunity to distinguish between these explanations by comparing the latency of the response. A genuine selectivity change predicts that the latency of the responses elicited by a combination of the stimuli would be similar to the typical latencies of MTL neurons, whereas activity-spreading mechanisms (for example, pattern completion), where one stimulus activates the representation of the next, predict a delayed activation by the preceding stimulus. For our MTL cells the mean latency during the screening sessions was 258±157 ms (mean±s.d.), consistent with previous estimates of latencies of human MTL cells^25^. In contrast, during the AL sessions the mean latency of response to the preceding stimulus was significantly longer (1,703±740 ms), in support of the hypothesis that new associations cause activity to spread from the representation of the preceding stimulus to the representation of the preferred stimulus. Our results do not exclude the possibility, however, that with prolonged training new chunks emerge and that they could be represented by pair-coding neurons.

Our results are consistent with previous neurophysiological evidence that the monkey MTL signals the formation of new associations between visual scenes and spatial locations^7^, and the retrieval of well-learned associations between visual stimuli^26^. At the same time, the present results go beyond these previous studies in two important aspects: our results demonstrate associative learning in human MTL neurons during the learning of a sequence of non-spatial stimuli; and on a relatively short timescale (in contrast to the typically much longer learning process in the previous electrophysiological studies in animals). In addition to the anticipatory activity during the ISI before the preferred stimulus, we also observed changes in the response elicited by the preceding stimuli themselves. However, these altered responses occurred only in the context of sequence learning (Supplementary Fig. 9), consistent with a similar observation for place cells^18^, and in accordance with the hypothesis that hippocampal neurons associate stimuli with the contexts in which they were experienced^27^. At the same time, our results thereby differ from previous studies demonstrating changes in neuronal selectivity that persisted outside the task context, as has been demonstrated for pair-coding neurons in the inferotemporal cortex and surrounding regions in monkeys^4^,^26^, and with fMRI in humans^12^. It seems likely that differences between brain regions, learning protocols and the type of learning explain these apparent discrepancies. For instance, one critical difference between our study and the studies in monkey pair-coding neurons is that pair-coding neurons are typically found in perirhinal cortex and area TE, whereas our learning effects were observed in the hippocampus and parahippocampal cortex. Furthermore, the monkey experiments used training protocols across many days, which presumably result in enduring changes in neuronal tuning, whereas our learning protocol lasted only a few minutes. There is also a notable difference in the type of learning between our study and the fMRI study mentioned above. We instructed the subjects to learn the sequence, whereas the previous fMRI study focused on incidental learning in a task that did not require them to learn the sequence. It is conceivable that explicit and implicit learning make different demands on MTL structures and result in different time courses of the memory traces. The precise conditions that determine the involvement of the different MTL structures and the time course of learning remain an exciting topic for future research.

The anticipatory activity observed by us differs from the influence of novelty and familiarity on MTL activity reported in previous studies. In two of these previous studies^28^,^29^, patients saw a set of novel images in a first recording session, which were then repeated, along with a new set of images, in a second recording session. In the first session, patients were asked to memorize the images, and in the second session they reported if these images were familiar. Single neurons in the hippocampus and parahippocampal cortex changed their response when a stimulus had become familiar, after a single stimulus presentation^19^,^20^. Interestingly, the influence of familiarity or novelty generalized across multiple visual stimuli. In a related study, Cameron *et al.*^30^ presented patients with word pairs that had to be remembered and later recalled. They found that the activity of hippocampal neurons during encoding could predict whether subjects would later remember the pairs, whereas entorhinal cortex activity was correlated with recall success. These studies provide support for the important contribution of human MTL neurons to memory processes. The current study provides evidence for a complimentary MTL contribution to memory by showing that neurons show predictive activity during the learning of arbitrary associations between unrelated stimuli that appear in a sequence. Furthermore, the sequence-learning effects were specific to the neurons’ preferred images, unlike the more generalized novelty and familiarity effects observed in previous studies. In addition, our learning effects appeared later, after 11 stimulus exposures, possibly reflecting the additional time needed to accumulate sufficient information to form an association between two unrelated stimuli.

We observed a learning effect in the MTL but failed to observe a similar effect in the temporal cortex. However, we cannot discount the possibility that other brain regions also support associative learning, and could even drive the changes in neuronal response observed here in the MTL. Indeed, as discussed above, several studies have shown that learning-related changes occur in area TE of inferior temporal (IT) cortex when monkeys learn to associate paired images^2^ or to classify stimuli into new categories^31^,^32^ although these changes in tuning occurred after more extensive training. In accordance with these results, human fMRI studies demonstrated that shape learning changes the spatial profile and amplitude of the BOLD response in extrastriate cortex^33^,^34^,^35^. Future studies could aim to determine the contributions of the MTL and IT to the learning of shapes and categories and also to the formation of new associations between these shapes. An important difference between IT and the MTL is that IT appears to be mainly concerned with the analysis of complex visual features, whereas the MTL is responsible for the transformation of visual information into mnemonic and conceptual representations.

Several studies of the human MTL have demonstrated the existence of ‘concept cells’^36^. These cells represent the meaning of a given stimulus, in a manner that is multimodal and invariant to different representations of that stimulus^37^,^38^, and a recent study demonstrated that MTL neurons can even code new associations between a person and a place within a few trials^39^. We have recently proposed that the ability of MTL neurons to encode associations between stimuli that occur together could account for the formation of concept cells^40^. Indeed, the statistical regularities of the world ensure that different representations of a given stimulus (for example, a person’s face and the sound of her name) co-occur frequently. We hypothesize that such statistical structure along with associative learning mechanisms, as revealed here, promotes the linkage of different forms of a given stimulus within the MTL to generate invariant representations.

Another unique feature of MTL neurons is that they are strongly modulated by attention^41^ and are also activated when subjects imagine a stimulus in the absence of any bottom–up visual input^42^, or bring to mind a past episode, for example, of a recently watched movie clip^43^. It seems likely that the influence of mental imagery and active rehearsal on the activity of MTL neurons is related to the predictive activity for the yet-to-be-shown stimuli in a fixed sequence. Thus, the predictive responses in the MTL during sequence learning may contribute to the neuronal mechanisms for the rehearsal of newly learned associations, causing predictive mental images of upcoming stimuli.

A key function of learning is to endow organisms with the ability to predict future events based on what was learned in the past. We suggest that the anticipatory responses of MTL neurons uncovered here may allow the human mind to predict upcoming events based on experiences that occurred only a few minutes ago.

## Methods

### Electrode implantation

Participants were eight patients (four female, age range 18–36 years) with pharmacologically intractable epilepsy undergoing a work up to determine eligibility for surgical therapy of the epilepsy at the SEIN (Stichting Epilepsie Instellingen Nederland)–VU Medical Center epilepsy surgery programme, Amsterdam, Netherlands. Patients were implanted with depth electrodes for 7–10 days for chronic seizure recording to localize the seizure focus for possible surgical resection^13^,^44^. All surgeries were performed by J.C.B. The Medical Ethics Committee at the VU Medical Center approved the studies. Written informed consent was obtained from all patients before participation. The electrode locations were based entirely and exclusively on clinical criteria (patient data of any kind necessary to determine a resection with highest probability to render the patient seizure free). Each electrode consisted of eight microwires from which we recorded single- and multi-unit activity and a ninth microwire that served as a local reference. The signal from the microwires was recorded using a 64-channel Neuralynx system, filtered between 1 and 9,000 Hz, sampled at 32 KHz. On average, each patient was implanted with 34±12 microwires. Participants sat in their hospital room and performed the experimental sessions on a laptop computer. All patients participated in the two types of experimental sessions described below.

### Spike detection and sorting

Spike detection and sorting were performed with wave_clus^45^. Briefly, the data were band pass filtered between 300 and 3,000 Hz and spikes were then detected with an automatic amplitude threshold. Spike sorting was performed with a wavelet transform that extracted the relevant features of the spike waveform. Clustering was performed using a super-paramagnetic clustering algorithm. As in a previous study^37^, the clusters were classified as single or multi-units (Supplementary Figs 1 and 2). Multi-unit clusters reflect the activity of several neurons that cannot be further differentiated due to a low signal-to-noise ratio. As in ref. 37, the classification between single and multi-unit was performed visually based on: (1) the spike shape and its variance; (2) the ratio between the spike peak value and the noise level; (3) the ISI distribution of each cluster; and (4) the presence of a refractory period for the single-units; that is, fewer than 1% of spikes in a 3-ms or smaller inter-spike interval.

### Number of neurons and their locations

Over the eight patients we recorded from 635 neurons (single and multi-units as defined above) in the left and right hippocampi, posterior temporal cortices, parahippocampal cortices and the left amygdala. Assuming an effect size of at least 0.5 we calculated that we needed 30 cells to achieve a statistical power of 0.75. We determined the selective neurons in the screening sessions (see below) with a paired *t*-test (*P*<0.05) that compared the response during the stimulus presentation period (0–1,000 ms following stimulus onset) with the preceding ISI (−500 to 0 ms before stimulus onset). Consistent with previous reports of selective neurons in the human MTL^37^, 80 (12.6%) of all neurons were selective to at least one image. A total of 56 of the selective neurons were targeted during AL, that is, their preferred stimuli were included in the subsequent AL sessions. In all, 32 of these 56 neurons were located in the right hippocampus, 8 in the left hippocampus, 2 in the left parahippocampal cortex (PHC) and 14 in the right posterior temporal lobe. These 42 neurons in the MTL structures (hippocampi and parahippocampal cortex) were analysed here (see Supplementary Fig. 5 for responses of the 14 temporal cortex neurons). All 42 MTL neurons that were identified as selective during the screening sessions were included in the AL analysis without any further preselection. Because of the small number of neurons in the PHC we were not able to statistically examine differences between hippocampal and PHC neurons.

### Screening sessions

On each day that the patient was available, she/he first performed a screening session during which the patient was presented with a large variety of different images (famous people, relatives, animals, landmarks, objects, etc.). Each image subtended 1.5° of visual angle and was presented at the centre of the screen. Images were presented for 1,000 ms, followed by an inter-stimulus interval of 500 ms. Each image was repeated eight times in a randomized order. Between 7 and 51 images were used in the screening sessions depending on the time span the patient was available. After the presentation of each image the patients performed a simple yes/no task, for example, ‘Did the picture contain a human face’? The exact question depended on the picture set. This task ensured that patients attended to the stimuli. Data from the screening sessions were rapidly analysed to determine which images were the ‘preferred’ images of the neurons. A neuron’s ‘preferred’ images were defined as those that elicited a significant (paired *t*-test, *P*<0.05) response during the stimulus presentation period (0–1,000 ms after stimulus onset) compared with the preceding ISI (−500 to 0 ms before stimulus onset).

### Associative learning sessions

Following the screening sessions, eight patients performed a total of 27 AL sessions, which were usually performed on different days. The AL sessions were designed using information from the screening sessions. In each AL session, subjects were presented with a sequence of 5–7 images, always in a predetermined order such that a given image, A, predicted the identity of the next image, B, and so on. Subjects were asked to remember the order of the stimuli in the sequence. Each stimulus was presented for 1,500 ms with an SI of 500 ms, resulting in individual trials of 2,000 ms (Fig. 1). The AL sessions were designed such that in each sequence of 5–7 images, at least one image was the preferred image of one of the recorded neurons. In the rare cases where the sequence consisted of more than one preferred image, only one of the preferred images was used in the subsequent analyses (in other words, the same neuron was never counted twice in an AL session). In addition, to be included in subsequent analyses, the stimulus immediately preceding the preferred stimulus in the sequence was required to be a non-preferred image (that is, it did not elicit a significant response during the screening session) for this neuron. The sequence was repeated continuously 60 times resulting in experimental sessions of ∼10–14 min, not including time spent by the subject on test trials. Twenty per cent of trials were ‘test’ trials in which, instead of being presented with the next image of the sequence, subjects were shown two images and asked to decide (by pressing one of two keys on the keyboard; Fig. 1c) which of the two would be the next image in the sequence. No constraint was placed on response times. Behavioural performance on the test trials is shown in Fig. 1d,e. The test trials were excluded from further analysis. There were no test trials in the first stimulus sequence presentation.

To further the impression of a sequence of images we used the following display arrangement: Each image was presented at the centre of the screen while three placeholders (empty grey squares) were presented to the left and right of the central image (Fig. 1b). At the end of the 1,500-ms presentation period, the central image was replaced by a grey placeholder and all seven grey squares moved one ‘step’ forward in a clockwise direction for the duration of the ISI, such that each placeholder eventually occupied the next placeholder position. At the end of the ISI the placeholder that now occupied the central position was replaced by the next image in the sequence. The viewer’s subjective impression at the end of the ISI interval was that the central image had been hidden, and then moved clockwise, while the central position was replaced by the next image in the sequence.

### Data analysis

For the individual cells shown in Fig. 2a,b, spikes were binned into 150-ms time bins to obtain the peri-stimulus time histograms. For individual cells, the smoothed response (black curve in Fig. 2a,b) was obtained by convolving the spike trains (that is, in 1-ms bins) with a Gaussian smoothing window with a s.d. of 100 ms (similar results were obtained with 50- and 70-ms smoothing windows). To compute the average across all cells (Fig. 2c), we smoothed the average response of each neuron with a 100-ms smoothing window as above, subtracted the average ISI activity (in the −500 to 0 ms interval before stimulus onset) before the ‘preceding’ stimulus, and averaged across all cells. This analysis corresponds to the baseline-corrected or equivalently, ISI-corrected firing rates. No further normalization was performed to obtain these figures. The only effect of this baseline correction is to set the activity during the preceding ISI to 0 (Fig. 2c). Without this correction the entire plot would simply be shifted upwards to some positive value corresponding to the preceding stimulus ISI activity, but would otherwise be unchanged. We also examined the influence of normalization before averaging (Supplementary Fig. 3). To normalize responses we calculated the mean firing rate of each cell for its preferred stimulus, across the screening and AL sessions. Firing rates were then normalized by this value to make all cells comparable. Normalization of responses before averaging gave almost identical results as averaging without normalization.

### Latencies

Instantaneous firing rate curves were calculated by convolving the spike trains (that is, in 1-ms bins) for each cell with a Gaussian smoothing window with a s.d. of 100 ms. The latency of response or the time of anticipatory activity was the first time point (in 1-ms bins) at which neuronal activity was significantly greater than the ISI (that is, the −500 to 0 ms interval before stimulus onset) before the preceding stimulus (paired *t*-test; *P*<0.05) for at least 100 ms in individual cells (Fig. 2a,b) and for 300 ms in the averaged response (Fig. 2c). Because of the smoothing the absolute latency values have a precision that depends on the size of the window. However, since the same smoothing is applied to the screening and AL responses, the comparison of anticipatory response latencies across these experimental conditions remains justified.

### Statistics

Significance of responses was determined by comparing average firing activity across the entire trial window (that is, −500 to 1,000 ms for the screening sessions and −500 to 5,500 ms for the AL sessions) at each time point to the average firing activity during the ISI (that is, −500 to 0 ms before stimulus onset) before the preceding stimuli, with a paired *t*-test. The responses marked significant (solid horizontal black lines in Fig. 2) correspond to time periods in which firing rates were significantly greater than the ISI activity (*P*<0.05), continuously for at least 100 ms for individual cells. In Fig. 2c (average across cells), the significant responses correspond to time periods in which firing rates were significantly greater than the ISI activity (*P*<0.05), continuously for at least 300 ms. Identical criteria were applied to data from the screening and AL sessions. Adjustments for multiple comparisons were made using the method proposed by ref. 46 to determine significant differences of time courses.

Two-tailed paired *t*-tests were only applied when the normality assumption was tenable. In all other situations the *t*-test was replaced by a non-parametric equivalent test (Mann–Whitney *U*-test). The sphericity assumption was verified for ANOVAs. If needed, we applied the Greenhouse–Geisser correction.

## Additional information

**How to cite this article:** Reddy, L. *et al.* Learning of anticipatory responses in single neurons of the human medial temporal lobe. *Nat. Commun.* 6:8556 doi: 10.1038/ncomms9556 (2015).

## Supplementary Material

Supplementary Information Supplementary Figures 1-9 and Supplementary References
