# Supplementary material for: Learning of anticipatory responses in single neurons of the human medial temporal lobe
Source: Nat Commun. 2015 Oct 9;6:8556. doi: 10.1038/ncomms9556 (PMC4617602; doi:10.1038/ncomms9556)
Supplement: Supplementary Information — Supplementary Figures 1-9 and Supplementary References [file ncomms9556-s1.pdf]

Supplementary Figures

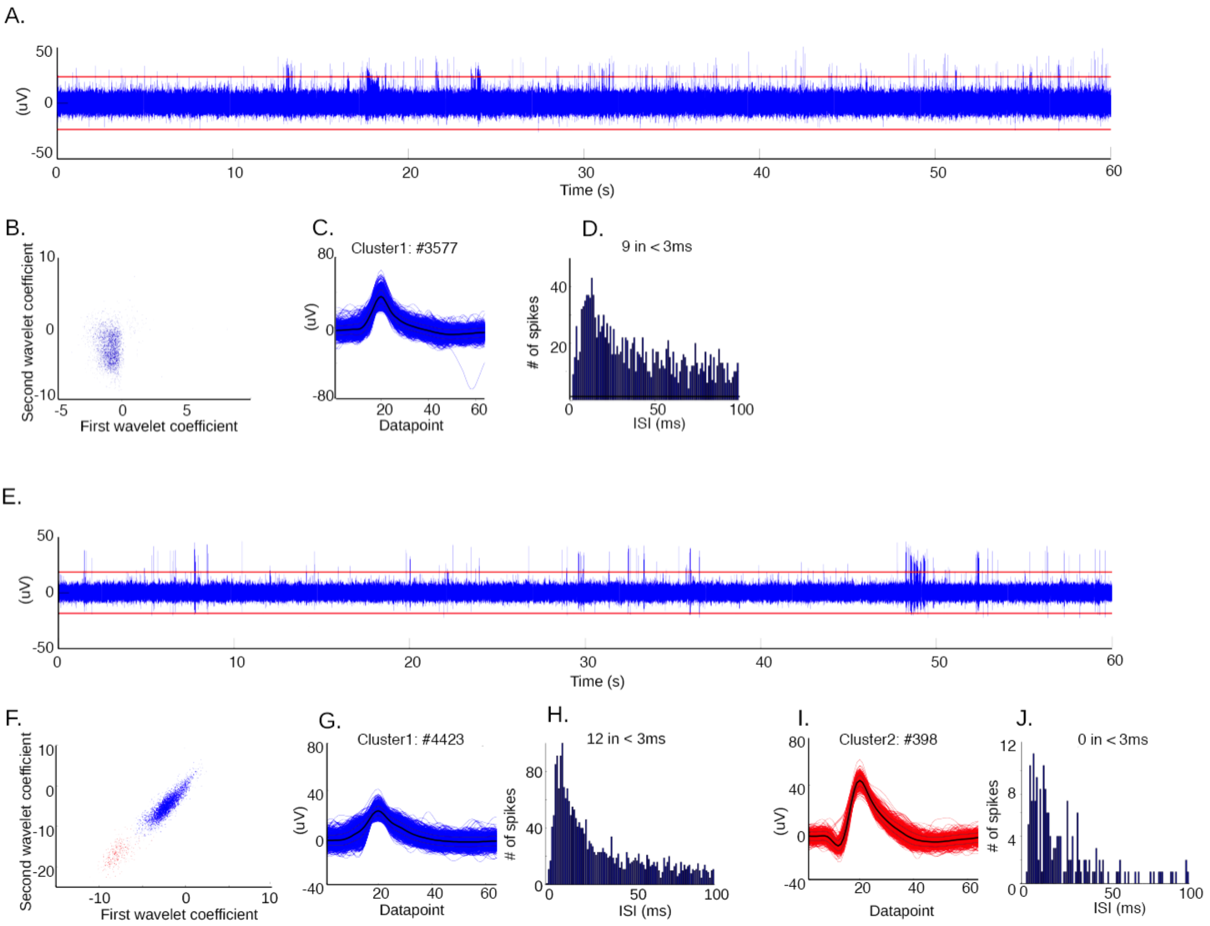

Supplementary Figure 1: Quality of spike sorting. Spike sorting procedure illustrated for electrodes in two patients in A-D and E-J. Spike sorting was performed with Wave_clus[2]. A, E: 60 second segment of data, bandpass filtered between 300-3000Hz. The red lines correspond to the thresholds used for spike detection, chosen to be five times the standard deviation (estimated from the median of the signal) of the band-pass filtered signal as described in[2]. B, F: Values of the first two wavelet coefficients. C, G, I: Waveforms of the sorted units. D, H, J: Inter-spike interval distributions. The cell illustrated in A-D is the same as the cell shown in Figure 2A and the red cluster in I, J corresponds to the cell shown in Figure 2B. C and G were classified as multi-units and I as a single-unit.

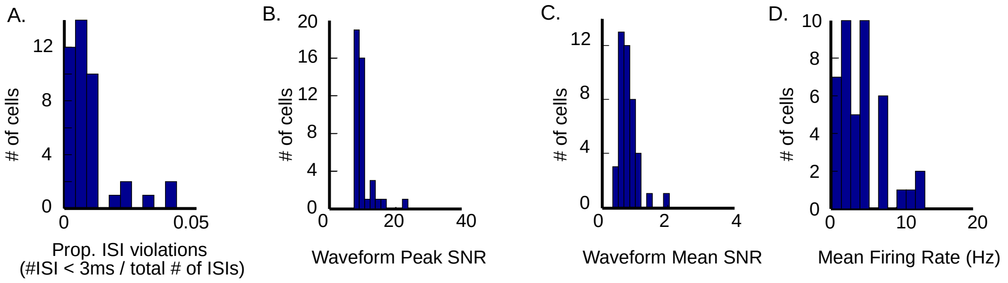

Supplementary Figure 2: Spike sorting quality for the 42 MTL neurons. A) Proportion of ISI violations (defined as the proportion of ISIs less than 3ms). B) Signal-to-noise ratio (SNR) based on the peak of the waveforms. C) Signal-to-noise ratio (SNR) based on the mean of the waveforms. D) Mean firing rates. In B) and C) the noise was quantified as the standard deviation of the band-pass filtered signal (including spikes), where the standard deviation was estimated from the median of the signal as in ref. [2]

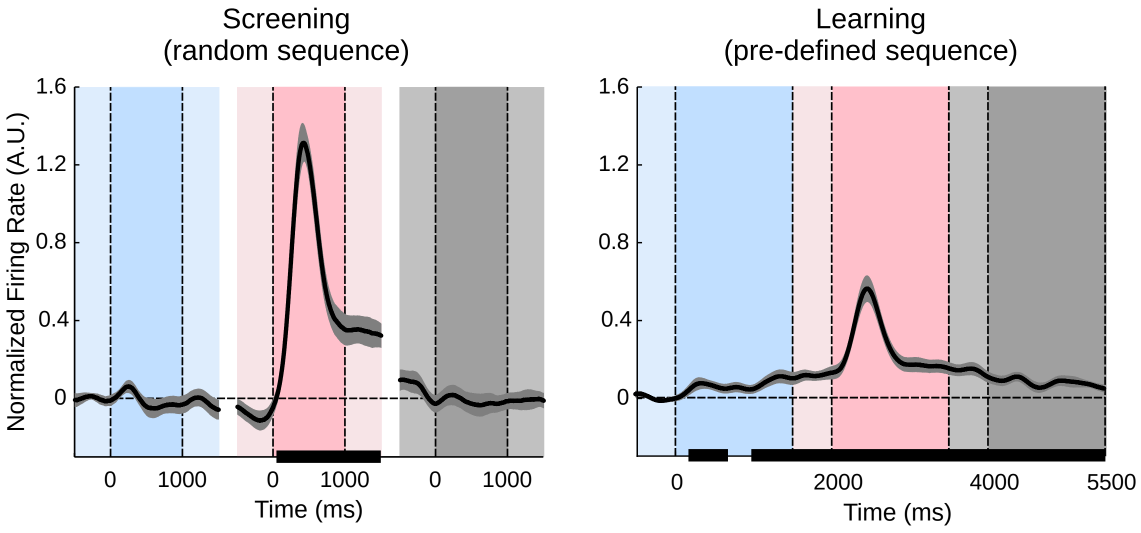

Supplementary Figure 3: Normalized average firing activity of the MTL cells during screening and AL. Figure format is similar to that in Figure 2C. To obtain normalized responses we first calculated the mean firing rate of each cell for its preferred stimulus, averaged across the screening and corresponding AL sessions. Firing rates in screening and AL sessions were then normalized by this value to make all cells comparable.

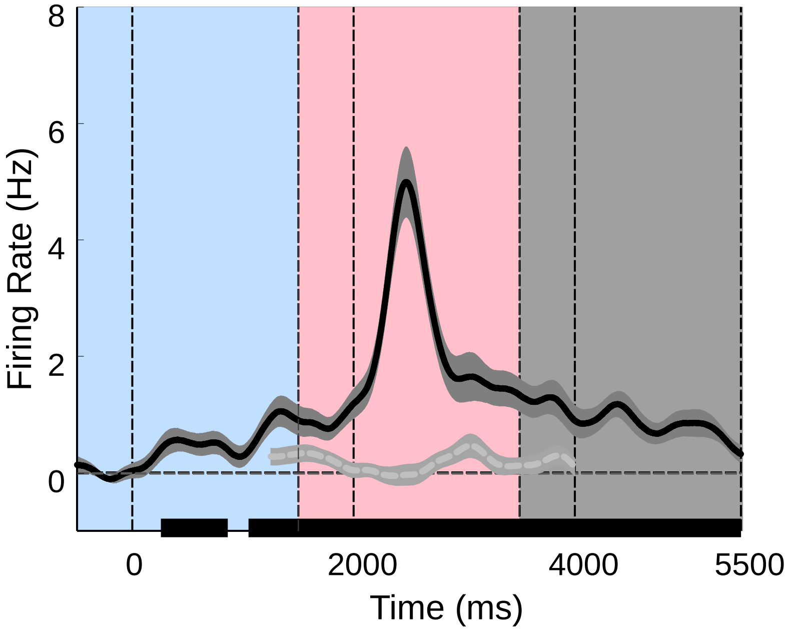

Supplementary Figure 4: The solid black curve represents the activity of the 42 MTL neurons during the presentation of the preceding, preferred and following stimuli (same data as in Figure 2C). The solid black horizontal line below the x-axis indicates periods with activity that differs significantly from that during the ISI (500ms window) prior to the preceding stimulus, continuously for at least 100ms (paired t-test, $p<0.05$). The dashed gray line is the response of the same neurons to the non-preferred stimuli (calculated in the 0-1500ms window after stimulus onset) presented during the sequence. This response was not significantly above the ISI activity prior to the preceding stimulus in any time interval (paired t-test, $p<0.05$).

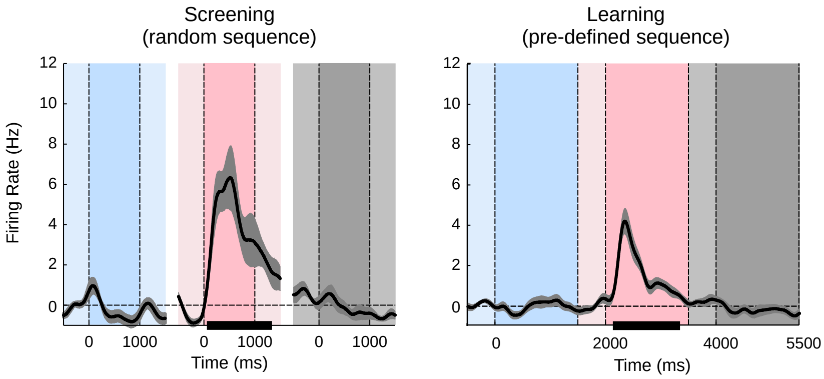

Supplementary Figure 5: Average activity of 14 posterior temporal cortex cells. Unlike the MTL cells, temporal cortex cells do not show an anticipation effect during AL (paired t-test, $p<0.05$). Figure format is similar to that in Figure 2C.

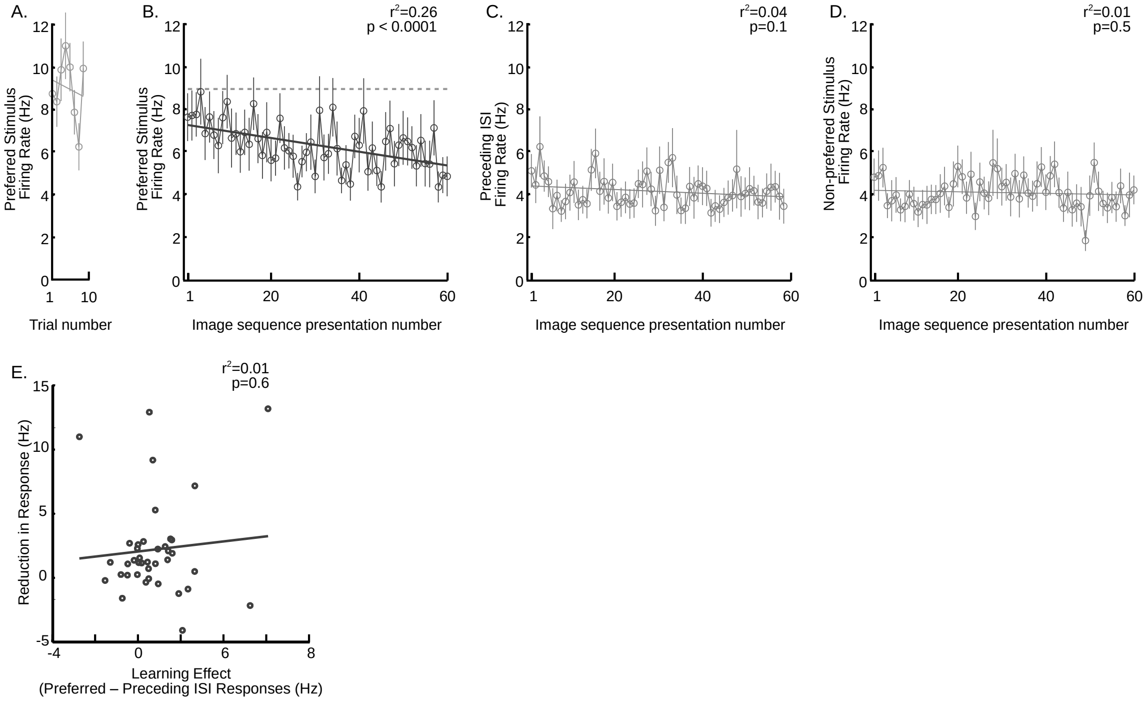

Supplementary Figure 6: Reduction in neuronal responses during the Screening and AL sessions.
A) Reduction in response during the screening sessions. The response of each cell to its preferred stimulus (during the 0-1000ms window following stimulus onset) as a function of trial number is shown here. The decrease in firing rate with trial number during the screening sessions was not significant (r^2=0.03, p=0.67).
B-E) As shown in Figure 2 B, C we observed a reduction in neuronal firing responses during the AL sessions compared to the screening sessions. Here we ask whether the reduction in response during AL is systematically related to image sequence presentation number (B), or whether it is a function of learning (E).
B) Preferred stimulus firing rates (increase in a 0-1000ms window following stimulus onset) as a function of image sequence presentation number in the AL sessions. A linear regression (diagonal black line) revealed that AL firing rates were negatively correlated with image sequence presentation number (r^2=0.26, p<0.0001). This consistent decrease in the firing rate is compatible with the habituation of neuronal responses. The dashed gray horizontal line corresponds to the mean firing rate during the screening sessions. A previous study has reported a reduction in firing rates as a function of trial number in human MTL neurons [1]. In comparison to a slope of ~3% per trial in Figure 2A of that study, we observed a reduction in firing rates of ~1% per trial. Thus, the habituation effect observed here is commensurate with the literature. However, the overall degree of habituation is stronger in our study because we presented the same stimulus on 60 trials, whereas the previous study repeated the same stimulus on only 6 trials.
C) Preceding ISI firing rates and D) non-preferred stimulus firing rates as a function of image sequence presentation number. The figure format is similar to that of B).
E) To investigate how the reduction in responses during AL varies with learning, we plotted the decrease in AL preferred stimulus baseline-corrected firing rates (i.e., the difference between the first 10 and last 10 stimulus presentations) as a function of the anticipatory learning effect for each cell. The anticipatory learning effect is defined as the preferred ISI response – the preceding ISI response. The regression analysis of reduction in firing rates with the learning effect was not significant (r^2=0.01, p=0.61).

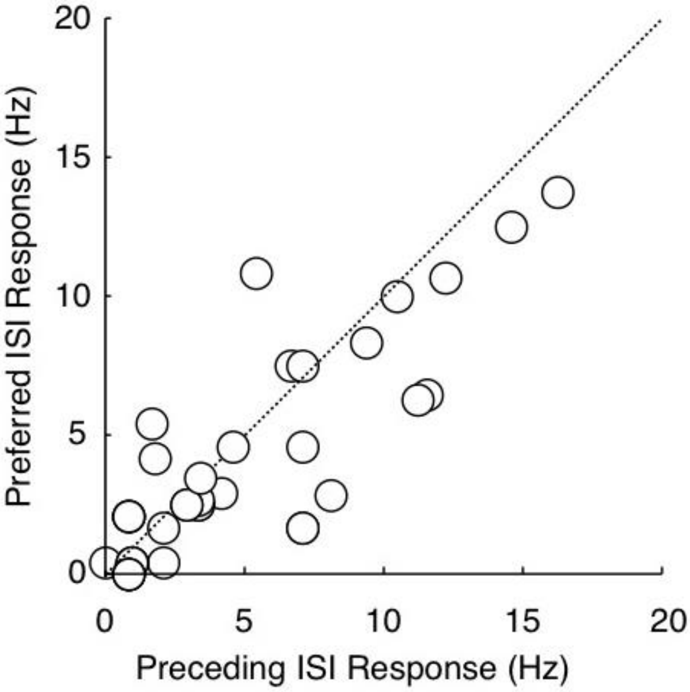

Supplementary Figure 7: Comparison of spiking activity during the ISI (500ms window before the stimulus) prior to the presentation of the preferred and preceding stimuli during the screening sessions, when the images were presented in a random order. During the screening sessions, the ISI activity before the preceding stimuli was slightly but significantly higher than that before the preferred stimuli ($p<0.05$; paired t-test). Note, however, that this difference in ISI activity during screening is in the opposite direction to the anticipatory learning effect observed during AL.

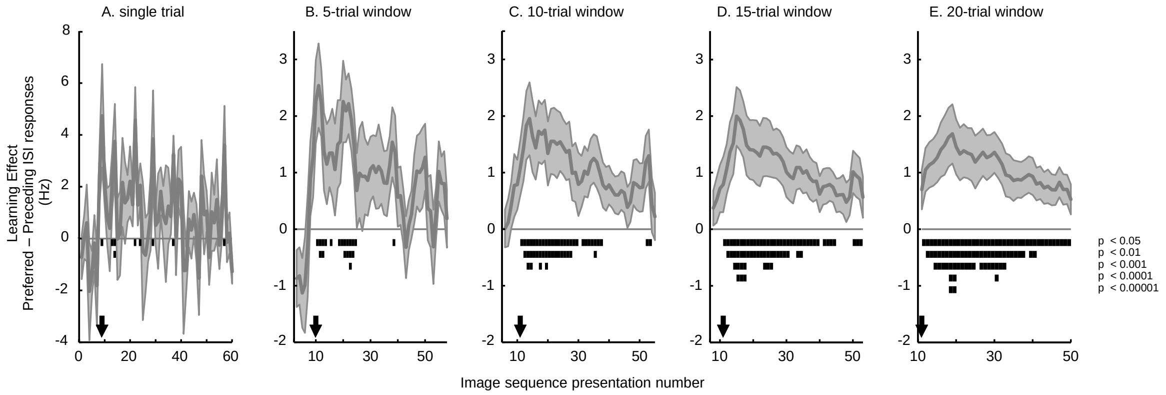

Supplementary Figure 8: The strength of the predictive activity on A) single trials and B-E) calculated with a running average of 5, 10, 15 or 20 trials, where on each trial *n* we considered the difference between the preferred and preceding ISIs based on a moving average of trials *n-m* to *n+m,* where *2m* is the size of the moving average window. Panel D (15-trial window) corresponds to Figure 3B. The solid black horizontal lines indicate which trials showed a significant learning effect, as computed by a non-parametric bootstrap procedure over 100,000 iterations. The black arrow indicates on which trial the difference between the preferred and preceding ISIs was first significant (p<0.05, non-parametric bootstrap proecdure).

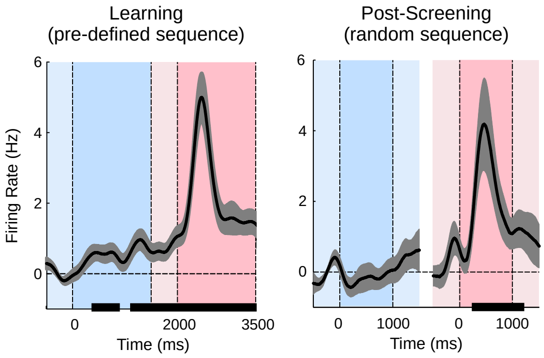

Supplementary Figure 9: Changes in neuronal tuning for the preceding stimuli do not persist in post-screening sessions after the AL sessions. For 27 of the 42 MTL cells the preceding and preferred stimuli were repeated in a subsequent post-screening session with images in a random order and outside a learning task so that the preceding stimulus no longer predicted the preferred stimulus. The left panel illustrates the predictive activity during the AL sessions for these 27 cells (same as Figure 2C but only averaging across 27 MTL cells). The right panel shows the responses of the same 27 cells during the later screening session. As expected, the anticipatory response did not occur during the later screening session.

Supplementary References

1 Pedreira, C. *et al.* Responses of human medial temporal lobe neurons are modulated by stimulus repetition. *Journal of neurophysiology* **103**, 97-107, doi:10.1152/jn.91323.2008 (2010).

2 Quiroga, R. Q., Nadasdy, Z. & Ben-Shaul, Y. Unsupervised spike detection and sorting with wavelets and superparamagnetic clustering. *Neural computation* **16**, 1661-1687, doi:10.1162/089976604774201631 (2004).
